# *Nannochloropsis oceanica*, a novel natural source of rumen-protected eicosapentaenoic acid (EPA) for ruminants

**DOI:** 10.1038/s41598-018-28576-7

**Published:** 2018-07-06

**Authors:** Susana P. Alves, Sofia H. Mendonça, Joana L. Silva, Rui J. B. Bessa

**Affiliations:** 1 CIISA - Centro de Investigação Interdisciplinar em Sanidade Animal, Faculdade de Medicina Veterinária, Universidade de Lisboa, Av. da Universidade Técnica, 1300-477 Lisboa, Portugal; 2 ALLMICROALGAE, Av. Eng. Duarte Pacheco 19, 9° piso, 1070-100 Lisboa, Portugal

## Abstract

We hypothesize that whole microalga biomass is a natural rumen-protected source of eicosapentaenoic acid (EPA, 20:5n-3) for ruminants. To test our hypothesis, we studied the ruminal biohydrogenation of EPA from two microalgae, *Nannochloropsis oceanica* and *Phaeodactylum tricornutum* using *in vitro* incubations with rumen fluid. A total mixed ration was incubated with: no EPA (control), EPA as free-fatty acid, *N. oceanica* spray-dried (SD), *N. oceanica* freeze-dried (FD), or *P. tricornutum* FD. The kinetics of EPA disappearance and of products formed during the 24 hours of incubation were evaluated, and complemented by deuterated-EPA incubation. Results showed that EPA metabolism from the *N. oceanica* was remarkably reduced compared with the *P. tricornutum* and free-EPA, and this reduction was even more effective with the *N. oceanica* FD. Our data also indicates that neither feed dry matter disappearance nor rumen microbial markers (branched-chain fatty acids and dimethyl acetals) were affected by EPA-sources. We reported for the first time the kinetics of EPA biohydrogenation class products and the unequivocal formation of 20:0 from EPA. Overall, *N. oceanica* shows a strong potential to be used as a natural dietary source of EPA to ruminants, nevertheless further studies are needed to verify its protection *in vivo*.

## Introduction

Public health nutrition guidelines recommend population-wide decreased saturated fatty acid (FA) and *trans* FA and increased polyunsaturated fatty acid (PUFA) consumption to lower the incidence of clinical metabolic diseases^1,2^. Indeed, the n-3 long-chain PUFA (n-3 LC-PUFA) including eicosapentaenoic acid (EPA, 20:5n-3) and the docosahexaenoic acid (DHA, 22:6 n-3) have been recommended due to their beneficial effects for humans, which includes anti-atherogenic, anti-thrombotic and anti-inflammatory properties^3^. Meat from land animals can be a source of n-3 LC-PUFA, particularly for populations where the consumption of fish and seafood is very low^4^. Nevertheless, in ruminants the content of n-3 LC-PUFA in meat is low (about 2 to 40 mg/100 g meat)^5^. Thus, there is a need to develop strategies to increase n-3 LC-PUFA in meat and milk^5,6^. The most efficacious approach is to feed animals with dietary n-3 LC-PUFA, like fish oil or more recently microalgae^6^. These strategies might have high success with monogastrics, however in ruminants most of the n-3 LC-PUFA included in the diet are hydrogenated in the rumen, producing saturated FA and a range of other FA intermediates^5,7^. This is due to the microbial processes that occur in the rumen, i.e. lipolysis of dietary esterified lipids followed by biohydrogenation of the released PUFA. The microbial biohydrogenation is the process responsible for the isomerization and hydrogenation of the FA unsaturated double bonds resulting in extensive metabolism of PUFA into saturated end-products limiting the escape of PUFA from the rumen. For that reason and considering the importance of consumption of ruminant derived products, several companies and research groups are trying to develop methods to protect n-3 LC-PUFA from the ruminal microbial population to increase the amount of PUFA available for deposition, elongation and desaturation in muscle and adipose tissue^8,9^.

Lipid rumen-protection approaches such as calcium salts, fatty acyl amides, aldehyde treatment, non-enzymatic browning, lipid composite gels, as well as novel interfacial cross-linking emulsions have been researched to resist to the ruminal microbial attack as was recently reviewed^8^. These approaches involve blocking the free FA carboxyl-end by forming calcium salts or fatty acyl amides, or involve encapsulation the lipid or FA inside a microbial-resistant shell. However, none of these methods were successfully applied in practice, either due to high cost, use of harmful products (e.g. formaldehyde encapsulation), or lack of consistency regarding rumen protection efficiency^8^. Thus, efficient rumen lipid bypass approaches are still needed.

There has been a growing interested in microalgae as a natural resource for biofuel, food and nutraceutical applications, including animal feed due to the nutritional and energetic value of microalgae constituents, particular n-3 LC-PUFA^10,11^. Microalgae are unicellular organisms in which the plasma membrane is protected by a complex cell wall that might display great diversity among species, strain and growing conditions. However, the most common constituents of microalgae cell walls are polysaccharides, including cellulose, lipids and proteins although some microalgae are also protected by an inorganic rigid wall composed of silica frustule of diatoms or calcium carbonate^12^. Mechanical, chemical and biological methods have been studied for cell wall disruption in order to extract the valuable cell components such as lipid and pigments^12^. From research of methods based on microbial enzymatic cell wall lysis, it became evident that several microalgae cell walls are somehow resistant to microbial attack^13^. Thus, we hypothesize that microalgae cell walls could partially resist ruminal microbial attack and protect PUFA from biohydrogenation. Although inclusion of fish oil or marine algae oils in ruminant diets have been shown to result in marginal PUFA enrichment in ruminant derived foods^14^, there are limited studies on the use of PUFA-rich microalgae biomass in ruminant diets. Therefore, this work aims to study if EPA-rich microalgae biomass has potential to be used as a natural source of rumen-protected n-3 LC-PUFA for ruminants. To this end, we evaluated *in vitro* the ruminal metabolism of EPA from two microalgae sources, the *Nannochloropsis oceanica* and the *Phaeodactylum tricornutum*, and compared that with the metabolism of free-EPA. However, because microalgae dehydration can cause damage to cells walls, the *N. oceanica* biomass was dehydrated by centrifugal spray-drying (SD) or freeze-drying (FD) processes. The effect of microalgae biomass on the ruminal biohydrogenation of C18 FA was also evaluated. In addition, the pattern of EPA biohydrogenation products formed from both free-EPA and microalgae were accessed and to confirm the origin of those products we also incubated EPA labeled with deuterium atoms.

## Results

### Influence of EPA-source on the ruminal metabolism and EPA disappearance

**Figure 1:**
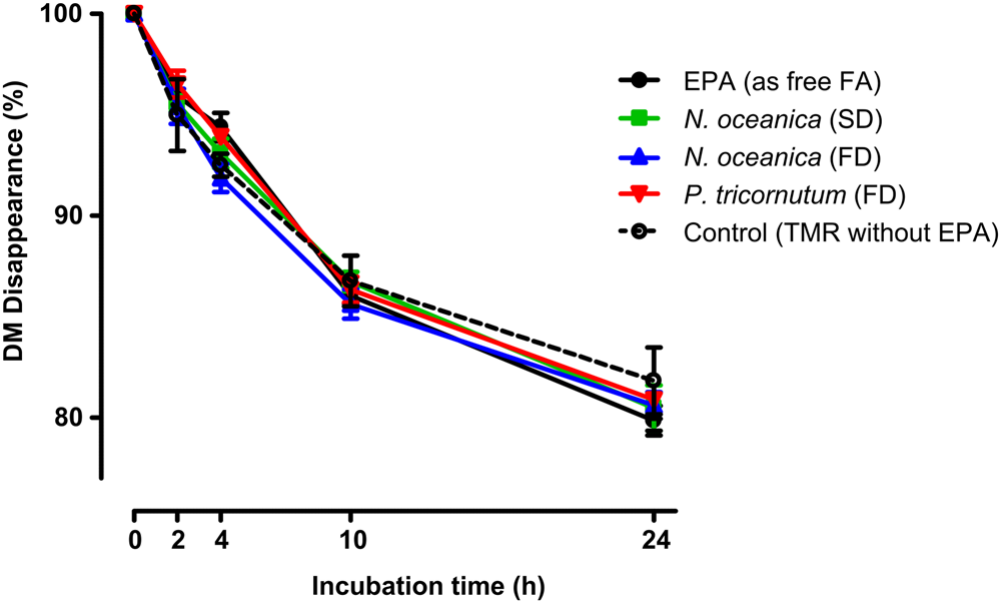
Dry matter (DM) disappearance through 0 to 24 hours of *in vitro* batch incubations with strained rumen fluid and a total mixed ration (TMR). The TMR was supplemented with: (1) EPA (as free FA), (2) *Nannochloropsis oceanica* spray-dried (SD), (3) *Nannochloropsis oceanica* freeze-dried (FD), (4) *Phaeodactylum tricornutum* freeze-dried (FD), or (5) any EPA addition (Control).

**Figure 2:**
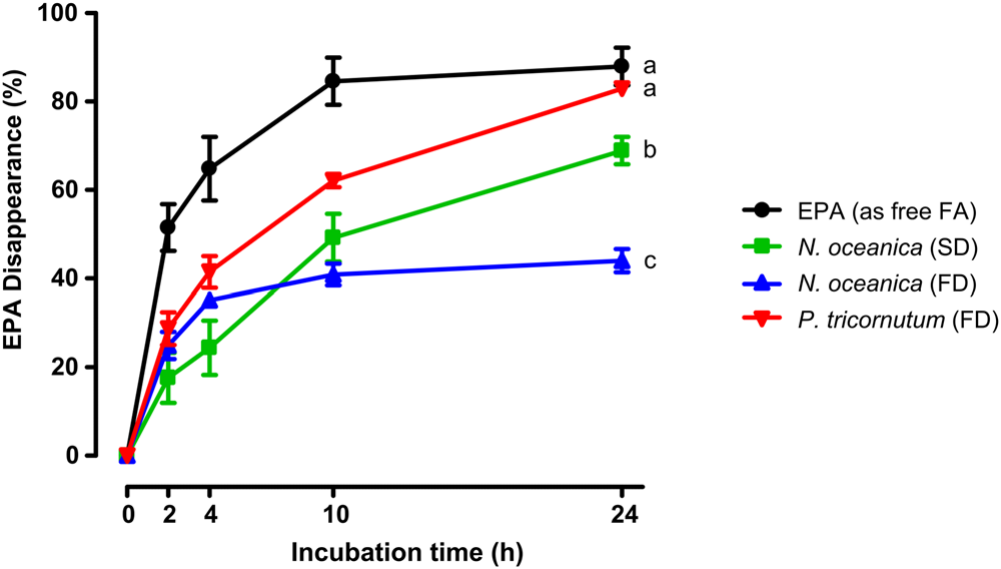
Disappearance (%) of EPA through 0 to 24 hours of *in vitro* batch incubations with strained rumen fluid and a total mixed ration (TMR). The TMR was supplemented with: (1) EPA (as free FA), (2) *Nannochloropsis oceanica* spray-dried (SD), (3) *Nannochloropsis oceanica* freeze-dried (FD), or (4) *Phaeodactylum tricornutum* freeze-dried (FD). Different letters (**a**–**c**) at 24 hours of incubation indicates significant differences (*P* < 0.05) between means.

Dry matter (DM) disappearance in tubes containing about 0.05 mg of EPA per mL of incubation medium added in the form of free-FA or microalgae biomass did not differ (*P* > 0.05) from control tubes (without EPA addition) during 0 to 24 hours of incubation (Fig. 1). However, considering the EPA-source we verified that EPA was metabolized at different rates in rumen fluid (Fig. 2). At 2 hours of incubation, the disappearance of EPA in tubes with the addition of free-EPA was already 51% while in tubes with *P. tricornutum* FD, *N. oceanica* SD and FD the EPA disappearance was only 29, 18 and 25%, respectively. At 24 hours of incubation the highest disappearance of EPA was observed in tubes with the addition of free-EPA or *P. tricornutum*, reaching 88 and 83%, respectively. The lowest disappearance was observed in tubes containing *N. oceanica* FD, reaching only 44% of EPA disappearance at 24 hours of incubation and the *N. oceanica* SD showed intermediate values, i.e. 69% of EPA disappearance.

The effect of EPA-sources on the FA with less than C18 carbon-atoms, as well as on the DMA composition after 24 hours of *in vitro* batch incubations with strained rumen fluid are presented in Supplementary Table S1. According to our data no effects of EPA-source were observed on the iso (i-), anteiso (a-) or odd-chain FA (*P* > 0.05), the only exception was the 15:0 that showed the highest quantity in incubation tubes with the *P. tricornutum*. Regarding the dimethyl acetal (DMA) composition, with the exception of *c*9-18:1, no significant effects *(P* > 0.05) of EPA-sources were observed on the other nine DMA detected in incubation tubes.

### EPA ruminal biohydrogenation

**Figure 3:**
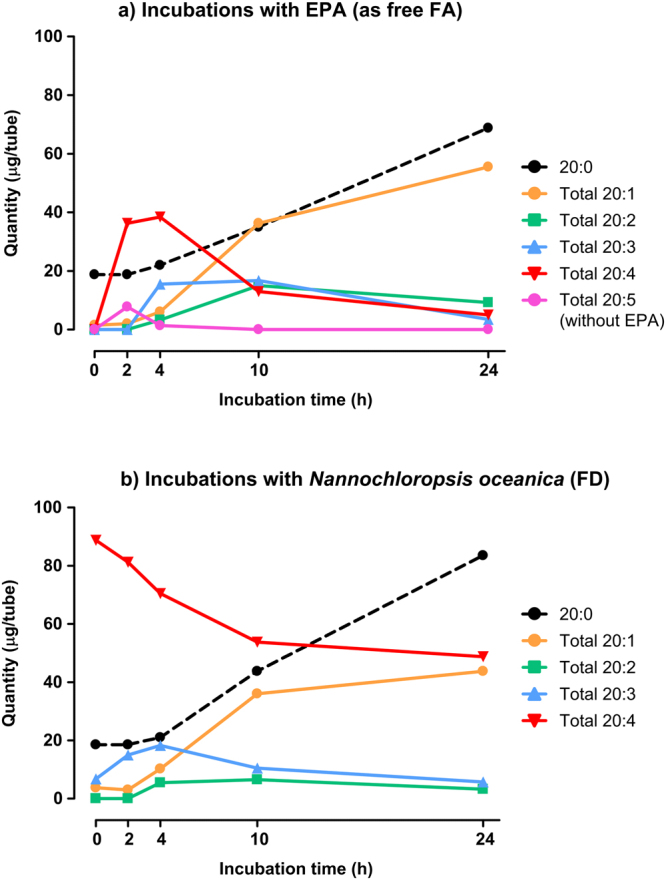
Quantity (µg/tube) of C20 fatty acids according to the number of double bonds through 0 to 24 hours of incubation with strained rumen fluid and a total mixed ration (TMR). The TMR was supplemented with free-EPA (**a**) or supplemented with *Nannochloropsis oceanic*a as FD (**b**). Total 20:5, includes one geometric isomer of EPA; Total 20:4, includes 20:4n-3, 20:4n-6 and other isomers of undetermined double bond position; Total 20:3, includes 20:3n-3 as the major isomer and other minor isomers of undetermined double bond position; Total 20:2, includes two isomers of undetermined double bond position; Total 20:1, includes at least 9 peaks identified as 20:1 isomers. Means and standard errors, as well as, linear, quadratic and cubic time polynomial contrasts are presented as Supplementary Table S2.

Figure 3 shows the kinetic curves of EPA biohydrogenation products formed during *in vitro* incubations with free-EPA or *N. oceanica* FD. The biohydrogenation products included 20:5, 20:4, 20:3, 20:2, 20:1 and the end product of rumen biohydrogenation, the 20:0. Identification of FA chain length as well as number of double bonds was achieved using GC-MS and also by comparison with published chromatograms of EPA isomers^15,16^. Complete characterization of EPA biohydrogenation intermediates is currently being carried out using multi-complementary techniques and will not be the focus of this work.

**Figure 4:**
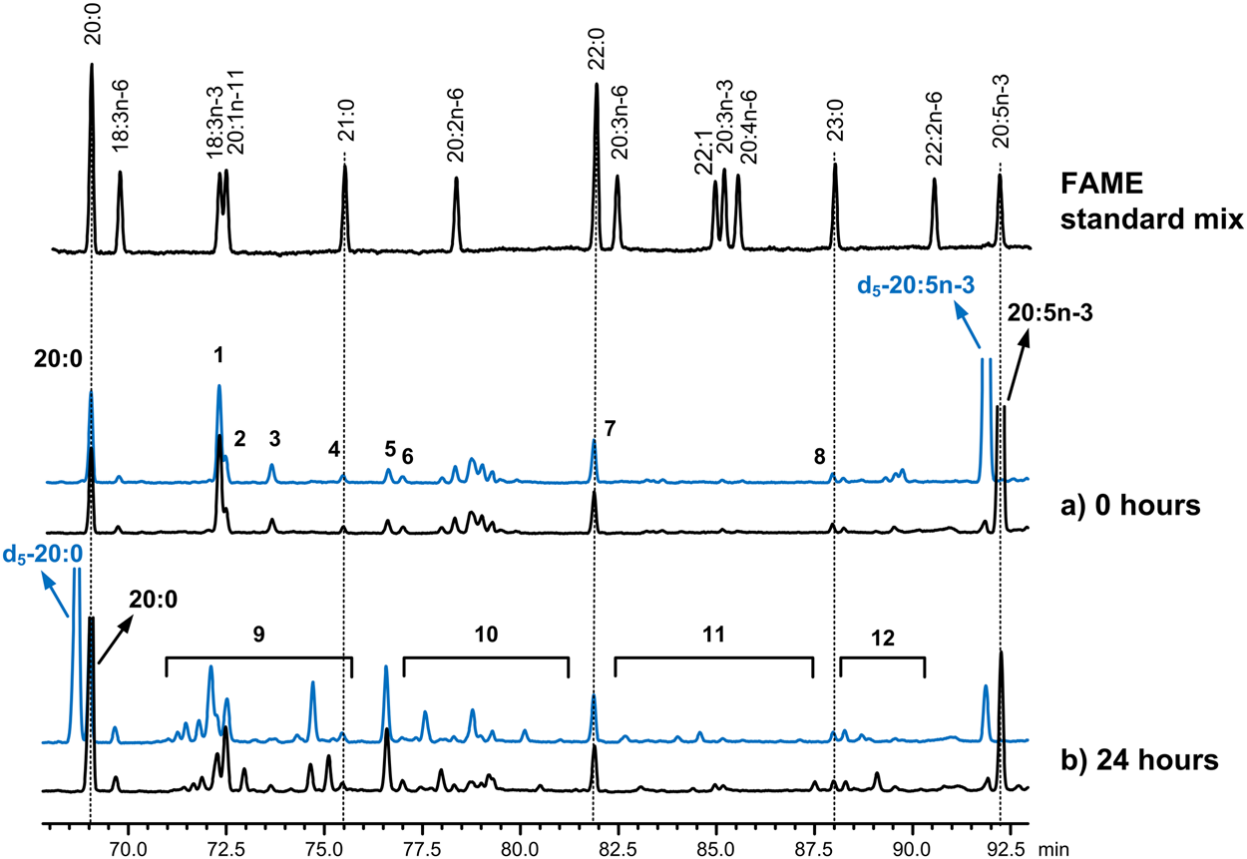
Partial GC-MS chromatograms from the region between 68 and 93 min from *in vitro* batch incubations with strained rumen fluid and a total mixed ration (TMR). The TMR was supplemented with (i) EPA (20:5n-3), black line, or (ii) deuterated EPA (d_5_-20:5n-3), blue line, at 0 hours (**a**) or 24 hours (**b**). Peak identification: 1, 18:3n-3; 2, 20:1n-11, 3, *cis*-9,*trans*-11-18:2; 4, 21:0; 5, dimethylester-12:0; 6, *trans*,*trans*-18:2; 7, 22:0; 8, 23:0; 9, 20:1 and d_5_-20:1 isomers region; 10, 20:2 and d_5_-20:2 isomers region; 11, 20:3 and d_5_-20:3 isomers region; 12, 20:4 and d_5_-20:4 isomers region.

Nevertheless, according to the Fig. 3a we verified that at 2 hours of incubation the major products formed from free-EPA were a geometric isomer of 20:5 (most likely a *trans* isomer, according to the chromatographic elution order^15,16^) and a few 20:4 isomers, resulting from hydrogenation of one double bond, while no 20:5 geometric isomers were detected in incubations with *N. oceanica* FD (Fig. 3b). The initial (0 hours) high quantity of 20:4 in incubations with the *N. oceanica* FD and its decrease over time derives from the 20:4n-6 present in the *N. oceanica* and its subsequent biohydrogenation. In both Fig. 3a,b the 20:3 products reached their maximum at 4 hours of incubation and minor quantities of 20:2 also started to be formed. At 10 hours of incubation the quantity of 20:4 isomers formed was greatly reduced while a greater accumulation of 20:1 isomers was observed. Finally, at 24 hours of incubation, it was observed a large increase of 20:0, the final product of EPA biohydrogenation. The formation of these C20 FA was confirmed through incubation with a deuterated isotope of EPA (19, 19, 20, 20, 20-d_5_ 20:5n-3, d_5_-EPA). Figure 4 shows GC-MS partial chromatograms of samples incubated with free-EPA or d_5_-EPA at 0 and 24 hours of incubation with rumen microbes. According to the Fig. 4 the d_5_-EPA and its biohydrogenation intermediates elute a few seconds before the non-deuterated EPA and products. The d_5_-EPA allowed distinguishing the 20:0 derived from EPA biohydrogenation or from that naturally present in the rumen or *de novo* formed. Hence, we verified that the 20:0 resultant from biohydrogenation only started to accumulate in the rumen after 4 hours of incubation with mixed rumen microbes (Supplementary Fig. S1). In addition, the linear, quadratic and cubic effects of EPA biohydrogenation intermediates produced during the 24 hours of incubation from each EPA-source are presented in Supplementary Table S2.

#### Influence of EPA-source on the metabolism of C18 FA

**Figure 5:**
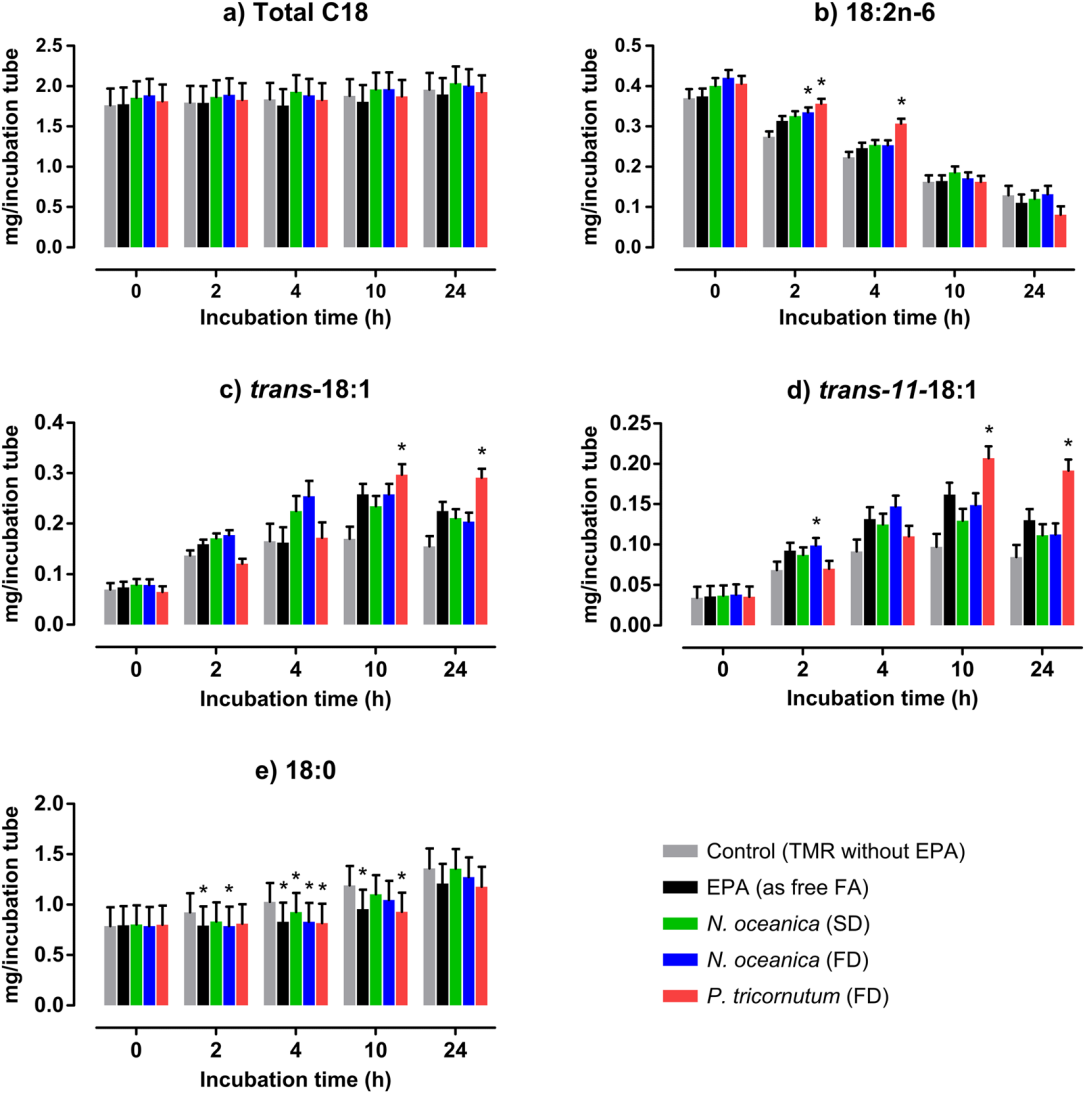
Quantity (mg/incubation tube) of Total C18 fatty acids (**a**), 18:2n-6 (**b**), *trans*-18:1 (**c**), *trans*-11-18:1 (**d**) and 18:0 (**e**) through 0 to 24 hours of *in vitro* batch incubations with strained rumen fluid and a total mixed ration (TMR). The TMR was supplemented with: no EPA source (Control), EPA (as free FA), *Nannochloropsis oceanica* spray-dried (SD), *Nannochloropsis ocanica* freeze-dried (FD), or *Phaeodactylum tricornutum* freeze-dried (FD). Significant differences (*P* < 0.05) between control (no EPA) and EPA-sources at each incubation time are presented with an asterisk (*).

Figure 5 shows the effect of EPA-sources on the metabolism of C18 FA compared with the control group (containing no EPA) at 0 to 24 hours of incubation with mixed rumen microbes. Independent of incubation time, the presence of free-EPA or EPA-rich microalgae did not affect (*P* > 0.05) the content of total C18 FA in incubation tubes compared with control (Fig. 5a). Nevertheless, some C18 FA differed (*P* < 0.05) from control at particular incubation times. The concentration of 18:2n-6 was higher (*P* < 0.05) in tubes with *P. tricornutum* at 2 and 4 hours of incubation compared to control, while in those with the *N. oceanica* the concentration of 18:2n-6 was only higher than control at 2 hours of incubation (Fig. 5b). Regarding the major 18:2n-6 biohydrogenation products, i.e. the *trans*-18:1 (Fig. 5c) and in particular the *trans*-11-18:1 (Fig. 5d), we observed that compared to control they only increased (*P* < 0.05) in incubation tubes with *P. tricornutum* at 10 and 24 hours. The exception was the *trans*-11-18:1 that at 2 hours of incubation its concentration was higher (*P* < 0.05) on incubation tubes with *N. oceanica* FD compared to control. Regarding the 18:0 its concentration was higher (*P* < 0.05) in control group compared with free-EPA and *N. oceanica* FD at 2 hours, with free-EPA and all EPA-rich microalgae groups at 4 hours, and with free-EPA and *P. tricornutum* at 10 hours of incubation with rumen microbes (Fig. 5e).

## Discussion

The rumen microbiota is responsible for metabolizing dietary lipids and limiting the amount of PUFA that is deposited in meat, milk and ruminant tissues^5,17^. Taking into consideration that most of the microalgae are protected by a complex cell wall^18^, we hypothesized that their cell walls could protect the dietary PUFA from the microbial attack in the rumen, and thus whole microalgae biomass could be used as a natural source of rumen-protected PUFA for ruminants. For this study, two microalgae rich in EPA were tested, the *N. oceanica* and the *P. tricornutum*, containing about 29% and 25% of EPA in total FA, respectively. Regarding the marine microalgae species characterized by EPA production the *Nannochloropsis* sp. and the *P. tricornutum* are the ones with the highest levels of EPA^19^. However, to the best of our knowledge there are no studies using these two microalgae in experiments with ruminants. It is well-known that in the rumen triglycerides are rapidly hydrolyzed by rumen microbes forming free-FA that are afterwards biohydrogenated forming saturared end-products and several FA intermediates^17^. Since biohydrogenation of PUFA only occurs if FA are in the free form, we compared the metabolism of EPA from *N. oceanica* and *P. tricornutum* with free-EPA. The amount of each microalga used in this study was selected to have similar concentrations of EPA in the incubation tubes (5 g EPA/kg TMR) and taking into consideration the future practical application *in vivo*. Despite the different quantities of algae biomass added to the incubation tubes no differences were observed for the DM disappearance compared to control (Fig. 1), which suggests that neither the algae biomass nor the quantity of PUFA in incubation tubes affected the microbial activity involved in rumen degradation. Moreover, as rumen microbial membranes are rich in branched-chain FA (mostly iso and anteiso) and plasmalogens (analyzed as DMA), both the iso- and anteiso-FA, as well as the DMA composition might be used as rumen microbial markers^20^. Consistent with the absence of effects on DM disappearance, no relevant differences were observed in the quantity of branched-chain FA and DMA after 24 hours of incubation between control and EPA-sources (Supplementary Table S1). This suggests that apparently microalgae did not inhibit the growth and activity of rumen bacteria. Our results are in line with the studies of Marrez *et al*.^21^, which found that total bacteria count was only negatively affected when more than 4% of dried *Nannochloropsis limnetica* was incubated with rumen fluid *in vitro*.

Surprisingly, our data show that EPA disappearance from *N. oceanica* was significantly lower compared with EPA disappearances from both *P. tricornutum* and free-EPA after incubation with rumen fluid for 24 hours. Also, no differences were observed for the EPA disappearance between *P. tricornutum* and free-EPA. These results suggest that *N. oceanica* is highly resistant to attack by ruminal microbes compared with *P. tricornutum*. Differences in cell wall structure and composition might explain these results. Indeed, earlier studies have reported that microalgae might differ on their susceptibility to microbial attack and that cell wall constituents are the major determinant of algal resistance^13,22^. *P. tricornutum* is a unicellular diatom belonging to the *Bacillariophyceae* class of algae, which in contrast with other diatoms microalgae presents a cell wall poor in silica and mainly composed of organic molecules, particularly, a sulphated glucuronomannan as the main polysaccharide^23^. The *Nannochloropsis* sp. are a phototrophic species from the *Eustigmatophyceae* class of algae that have been described to have a bilayer structure consisting of a thick cellulosic inner wall protected by an outer hydrophobic algaenan layer of highly saturated and long-chain aliphatic with ether cross-links^12,24^. So, *Nannochloropsis* sp. are very robust and refractory to cellular disruption, and among 6 individual *Nannochloropsis* species, the *Nannochloropsis oceanica* was reported to have the thickest cell walls^25^. Considering these complex cell wall characteristics, several researchers have been exploring efficient and cost-effective methods for *Nannochloropsis* sp. cell wall disruption for lipid or pigment extraction^25–27^. Also, studies on the accessibility of proteins and lipids from *Nannochlorosis oculata* with gastric lipases showed that it is necessary to disrupt the cell walls to make the lipids accessible to the digestive enzymes^28^. Thus, we suggest the *N. oceanica* with its cellulosic wall protected by an outer algaenan layer might have blocked ruminal microbial enzymes which act on lipids, and so protected EPA from biohydrogenation in the rumen, while *P. tricornutum* cell wall are less resistant to microbial attack.

Despite the significantly lower EPA disappearance from the *N. oceanica* compared with both *P. tricornutum* and free-EPA when incubated with rumen fluid for 24 hours, we also observed differences in EPA disappearances between the *N. oceanica* dehydrated by SD and FD (Fig. 2). Indeed, the EPA disappearance from the *N. oceanica* FD was 25% lower compared with the *N. oceanica* SD. Remarkably, the *N. oceanica* FD showed less than 50% EPA disappearance after 24 hours incubation with rumen fluid. These differences could be explained by the algae slurry drying method used to prepare the microalgae biomass. Spray drying involves liquid atomization, gas/droplet mixing and drying from liquid droplets. The atomized droplets are sprayed downward into a vertical tower through which hot gases pass downward. Drying is completed within a few seconds, however it might rupture intact cells due to high-pressure atomization^29^. Some authors also indicate freeze-drying can cause damage to cells, as intracellular water expands upon freezing^12,30^, however, other authors shown freeze-drying appears to be an effective method for preserving the vitality of algal strains^31^. In a study comparing the microstructure of spray-dried and freeze-dried microalgae Chlorella and Spirulina, the authors demonstrated that the method of drying, as well as cellular composition and concentration, temperature and drying times affected the external morphology of the resulting microalgae powders^30^. In fact, while spray-dried microalgae formed individual spheres with a void space in the center, freeze-dried algae formed sheets of cells that adhered together in a linear fashion^30^. In our study the differences observed between *N. oceanica* SD and FD on the EPA disappearance could probably be explained by differences in cell morphology or cell wall disturbances resultant from the drying method, allowing the *N. oceanica* SD to be more accessible to the rumen microbial enzymes. Thus, it seems that freeze-drying the *N. oceanica* slurry still preserves its cell walls, and when incorporated in ruminant diets, it has great potential to promote the bypass of lipids from the rumen. It is expected that after passage to the abomasum, the low milieu pH will promote algae cell disintegration allowing the digestion and absorption of EPA and other nutrients in the intestine and further deposition into ruminant tissues. Nevertheless, further research is needed to evaluate *in vivo* if the inclusion of whole *Nannochloropsis* sp. biomass in ruminants’ diet could effectively enhance the content of EPA in meat and milk.

The majority of studies using marine microalgae as a dietary source of PUFA for ruminants do not use whole dried microalgae biomass but extracted algal oil concentrated products, mostly due to the increased development of microalgae oil production and availability of commercial algal oils^19^. In ruminants, marine oils tested *in vitro*^32^ or included in diets with the intent to enrich meat^33,34^ or milk^35–39^ with PUFA, are mostly rich in DHA and extracted from *Schizochytrium* sp. and *Isochrisys* sp. In some experiments, the dietary marine oils increased the content of n-3 LC-PUFA in meat or milk compared with the control diet (without DHA-oil), however, none of these studies compared marine oils with the whole algae dried biomass. More limited are studies using microalgae rich in EPA, particularly those investigating EPA metabolism or incorporation into ruminant tissues. To the best of our knowledge, only one study evaluated FA ruminal biohydrogenation of six species of microalgae biomass, including the *Nannochloropsis granulate*, also rich in EPA. After *in vitro* incubation with ruminal fluid of the whole microalgal for 24 hours, the authors found that the quantity of EPA did not decreased, which supports the potential of the *Nannochloropsis* sp. as a source of natural protected-EPA for ruminants.

In the present study we also evaluated the pattern of products formed during EPA biohydrogenation in the rumen, and to better evaluate that, we incubated rumen fluid with EPA labeled with five deuterium atoms (d_5_-EPA) located on the terminal carbons (C19 and C20). Despite the few reports studying the biohydrogenation of EPA in the rumen^40–43^, to the best of our knowledge this is the first study using isotope labeled standards. The analysis of the d_5_-EPA biohydrogenation products showed that apparently there is no evidence of chain-shortening as also verified for the DHA biohydrogenation^7^. Moreover, because rumen microorganism are known to synthesize long-chain FA^44,45^, particular branched and saturated FA, rumen incubation with d_5_-EPA also allowed us to distinguish qualitatively the 20:0 formed from EPA biohydrogenation, *de novo* synthesized by rumen bacteria or already present in incubation medium. According to our results, the 20:0 formed from biohydrogenation of EPA only started to accumulate in incubation tubes after 4 hour of incubation with rumen fluid (Supplementary Fig. S1). Indeed, the kinetic curves of the products formed when free-EPA or microalgae were incubated with rumen fluid for 2, 4, 10 and 24 hours (Fig. 3) shows a great increase in the amount of 20:0 in tubes at 4 hours of incubation. To our knowledge this is the first study reporting the kinetic curves of EPA biohydrogenation intermediates formed in the rumen, and they confirm that EPA is successively hydrogenated to 20:4 > 20:3 > 20:2 > 20:1 > 20:0. We also verified that during the first hours, limited geometric isomerization of a *cis* double-bond occurs most likely to form a *trans*-EPA, but no 20-carbon FA containing a conjugated double bond were detected in our conditions. However, no geometric isomerization seems to occur when the source of EPA was *N. oceanica*, which suggests different rates on EPA biohydrogenation. In fact, due to the low EPA disappearance from the *N. oceanica* source, its metabolism is expected to occur at a lower rate, and possibly the appearance of the geometric isomer could be observed between 4 to 10 hours of incubation and so it was not detected in our conditions.

The major ruminal biohydrogenation pathways of C18 FA, e.g. linoleic (18:2n-6) and linolenic (18:3n-3) acids, involve initial positional isomerization with the formation of conjugated FA followed by successive hydrogenations^17^. Despite this, distinct biohydrogenation pathways of n-3 LC-PUFA compared to the C18 have been described. Kairenius *et al*.^46^ suggested that the ruminal biohydrogenation of DHA involves reduction of the *cis* double bond closest to the carboxyl group as the first step, but recently two new transient conjugated DHA products were identified, suggesting that the initial biohydrogenation of DHA was analogous to that of C18 FA^7^. Regarding the ruminal biohydrogenation pathways of EPA, little work has been done to clarify that so far, however, our results suggest that on the contrary to what is known for C18 FA and DHA, the biohydrogenation of EPA in the rumen does not appear to involve the formation of conjugated double bonds. Kairenius *et al*.^46^ also suggested that biohydrogenation of EPA involves reduction of *cis* double bonds and accumulation of several C20 intermediates, but their proposal resulted from analysis of omasal digesta after cows fed grass based-diets were supplemented with fish oil. Another study with anaerobic bacteria strains found that *Clostridium bifermentans* can convert EPA into several non-methylene interrupted FA with a *trans* double bond at carbon-7 with no evidence of the formation of C20 conjugated FA^42^. Thus, several pathways with different rates might be involved in EPA metabolism in the rumen.

From several *in vitro*^32,47^ and *in vivo*^35,38–40^ studies it is well accepted that dietary EPA and DHA affect the biohydrogenation of 18:2n-6 and 18:3n-3 in the rumen. Indeed, LC-PUFA seem to inhibit the reductase enzymes in ruminal microorganisms, which are responsible for the final hydrogenation of *trans*-18:1 to 18:0 causing the accumulation of *trans* C18 FA intermediates^47^. Therefore, several researchers have been supplementing ruminant diets with fish oil or marine algal-oils rich in n-3 LC-PUFA as a way to decrease the content of 18:0 and increase the content of C18 FA with potential health benefits (i.e. *trans*-11 18:1 and *cis*-9, *trans*-11 18:2) in milk and meat^14^. In our study, little effect of EPA-source was observed on the C18 FA compared to control (Fig. 5). Nevertheless, the *P. tricornutum* was the microalgae that induced more variations on the quantity of C18 FA compared with control, indeed there was an accumulation of total *trans*-18:1 isomers, in particular the *trans*-11 18:1, at 10 and 24 hours of incubation compared with control. The effect of *P. tricornutum* on the biohydrogenation of C18 FA might be related with the lower resistance of its cell walls to microbial attack. However, as no significant differences were observed on the C18 FA between control and free-EPA, we suggest that maybe other non-FA components from *P. tricornutum* had inhibited the microbial enzymes responsible for hydrogenation of *trans*-18:1 to 18:0 and not the EPA or its biohydrogenation products.

The inclusion of fish oil or marine algal-oils in diets of dairy ruminants as a source of n-3 LC-PUFA have been associated with milk fat depression, mainly due to the formation of specific biohydrogenation intermediates that inhibit milk fat synthesis^48^. *Trans*-10, *cis*-12 18:2 is the intermediate shown unequivocally to inhibit milk fat synthesis, however in animals supplemented with fish oil rich in EPA and DHA, the presence of *trans* C20 and C22 biohydrogenation intermediates originating from the rumen have been suggested to contribute directly or indirectly to milk fat depression^48^. Nevertheless, the effects of supplementing microalgae biomass to ruminants on the biohydrogenation of C18 FA, as well as its effects on milk fat depression have been little studied. In fact, the only study available seems to be with lactating dairy cows fed *Aurantiochytrium limacinum* biomass, a DHA-rich microalga, where no effects on milk fat depression, as well as on health and productivity parameters, were found^49^. As far as we know there are no studies of the inclusion of *P. tricornutum* or *N. oceanica* biomass in dairy ruminants. Nevertheless, considering the likely protection of *N. oceanica* cell walls to EPA, less C20 biohydrogenation intermediates are produced in the rumen and thus less effect might be expected on milk fat synthesis inhibition. Hence, future work should be done to study the potential of *N. oceanica* as a natural source of EPA for dairy ruminants and its effects on animal health and productivity parameters.

## Methods

### Standards and microalgae

The 20:5n-3 standard (>98% purity) as free FA and the labeled isotope standard d_5_-20:5n-3 (*cis*-5, 8, 11, 14, 17-eicosapentaenoic acid 19, 19, 20, 20, 20-d_5_; 98% atom %) were purchased from Sigma-aldrich (St. Louis, MO, USA). The two microalgae were produced at the microalgae industrial scale facility in Pataias (near Leiria, Portugal) and supplied by the company ALLMICROALGAE. The microalgae were chosen considering their high content in EPA and their availability. The *Nannochloropsis oceanica* was provided dehydrated by centrifugal spray-drying (SD) or as a slurry mass that was further freeze-dried (FD) at FMV laboratory. The *Phaeodactylum tricornutum* was only provided as a slurry mass, which was dehydrated by FD prior to the *in vitro* batch incubations with rumen fluid. The FA composition of the two microalgae is presented in Table 1.

**Table 1:** Fatty acid content (mg/g DM) and composition (g/100 g total fatty acids) of microalgae.

Item	*Nannochloropsis oceanica*	*Phaeodactylum tricornutum*
Total FA	120	55
14:0	4.4	9.3
16:0	20.0	18.4
16:1n-7	26.5	14.2
16:2n-4	0.2	3.5
16:3n-4	0.2	8.8
16:4n-1	n.d.	5.1
18:0	0.4	1.8
18:1n-9	3.9	0.5
18:2n-6	3.3	2.7
18:3n-3	0.8	0.2
18:4n-3	n.d.	0.6
20:0	0.1	0.2
20:3n-6	0.8	n.d.
20:4n-6	7.5	0.6
20:5n-3 (EPA)	28.9	24.6
22:0	0.3	0.5
22:6n-3	n.d.	1.6
24:0	n.d.	2.6
Other^1^	2.7	4.8

^1^Other fatty acids for *N. oceanica* include 10:0, 12:0, 14:1, 15:0, 17:0, 17:1, *c*11-18:1, 18:3n-6, 20:2n-6, 20:3n-3, and 20:4n-3 and for *P. tricornutum* include 12:0, 15:0, 16:1, *c*11-18:1, 18:3n-6, 21:0, 20:4n-3, and 24:1. n.d. - not detected.

### *In vitro* batch Incubations

The experimental animal procedures were approved by the Ethical and Animal Well-Being Commission (CEBEA) of the Faculty of Veterinary Medicine, University of Lisbon, Portugal (Protocol FMV/CEBEA 007/2016). All methods and procedures were performed following the established guidelines from this institution and following compliance guidelines of European Union (Directive 86/609/EEC). Additionally, authors S Alves and RJB Bessa hold a FELASA (Federation of European Laboratory Animal Society Associations) grade C certificate that enables designing and carrying out animal experimentation under European Union regulations.

For the *in vitro* batch incubations about 500 mL of ruminal fluid was collected from two fistulated sheep before the morning meal and immediately transferred to the laboratory in a thermostatic box at 39 °C. The pH of ruminal fluid was measured immediately (Digital pH meter; JP Selecta S.A, Barcelona, Spain) and ranged from 5.36 to 6.41 throughout the study. Rumen fluid was directly strained through four layers of cheesecloth and diluted (1:4, v/v) in the medium of Goering and Van Soest^50^ under CO_2_ flux. The ruminal buffered solution (6 mL) was distributed into Hungate tubes containing about 60 mg of a commercial total mixed ration (TMR) - Control group, or the TMR supplemented with: (1) 20:5n-3 (EPA) as free FA (12 µL of 20:5n-3 solution in methanol); (2) 9 mg of *N. oceanica* dehydrated by SD; (3) 9 mg of *N. oceanica* dehydrated by FD; or (4) 22 mg of *P. tricornutum* dehydrated by FD. The quantities of the 20:5n-3 standards and of each microalga were adjusted to have a concentration of 0.05 mg/mL EPA in incubation medium. The ethanol in the group of free-EPA was dried under a stream of nitrogen previous to the addition of the TMR. The TMR contained dehydrated alfalfa (700 g/kg), wheat grain (105 g/kg), soybean meal (110 g/kg), and minerals as premix (25 g/kg). The chemical composition of the TMR was 902 g/kg DM, 175 g/kg DM of crude protein, 113 g/kg DM of starch, 81 g/kg DM of ether extract and 213 g/kg DM of crude fiber. For batch incubations, Hungate tubes were filled with CO_2_ and closed with a butyl rubber stopper and screw cap, then tubes were incubated in a water bath (Unitronic, J.P. Selecta, Barcelona, Spain) for 0, 2, 4, 10, and 24 hours at 39 °C with gentle agitation (40 rpm). After incubation, tubes were directly frozen and stored at −20 °C. The allocation of tubes to the treatments, incubation time, order of filling with buffered ruminal fluid, and position in the water-bath were randomized. The incubation procedure was replicated over 4 consecutive weeks with one tube per treatment and time, except for the experiment with the labeled d_5_-20:5n-3 that was only performed one time. Samples were freeze-dried (ScanVac CoolSafe, LaboGene ApS, Lynge, Denmark), and stored at −20 °C until analysis.

### Fatty Acid Analysis

Fatty acid methyl esters (FAME) and dimethyl acetals (DMA) were prepared by direct transesterification with sodium methoxide (0.5 M) in methanol followed by the addition of hydrogen chloride (1.25 M) in methanol^20^. Methyl nonadecanoate (1 mg/mL) was added as internal standard. Fatty acid methyl esters and DMA were analyzed by gas chromatography with flame ionization detection (GC-FID) using a Shimadzu GC 2010-Plus (Shimadzu, Kyoto, Japan) equipped with a SP-2560 (100 m × 0.25 mm, 0.20 µm film thickness, Supelco, Bellefonte, PA, USA) capillary column. The chromatographic conditions were as follow: injector and detector temperatures were set at 250 °C and 280 °C, respectively; helium was used as the carrier gas at 1 mL/min constant flow; the initial oven temperature of 50 °C was held for 1 min, increased at 50 °C/min to 150 °C and held for 20 min, increased at 1 °C/min to 190 °C and then increased at 2 °C/min to 220 °C and held for 40 min. Identification of FAME and DMA were achieved by electron impact mass spectrometry using a Shimadzu GC–MS QP2010 Plus (Shimadzu) and according to published chromatograms^20^. The GC-MS chromatographic column and the GC conditions were similar to the GC-FID analysis. Additional mass spectrometer conditions were as follow: ion source temperature, 200 °C; interface temperature, 240 °C; emission voltage, 70 eV. Amounts of FA and DMA in incubation tubes were expressed as milligrams or micrograms per flask and determined using the internal standard, assuming direct proportionality between GC-FID peak area and compound weight.

### Calculations and Statistical Analysis

The proportionate disappearance of the EPA during 0, 2, 4, 10 and 24 hours of incubation were calculated as follow:

$${\rm{Disappearance}}\,( \% )=[({{\rm{EPA}}}_{{\rm{0}}}-{{\rm{EPA}}}_{{\rm{t}}})/{{\rm{EPA}}}_{{\rm{0}}}]\times {\rm{100}}$$

where, EPA is expressed in mg per tubes at time 0 (EPA_0_) and time t (EPA_t,_ t = 2, 4, 10, 24 hours).

EPA disappearances at each incubation time were analyzed using the MIXED procedure of SAS and considering the weekly runs as random block and the EPA-source as the single fixed effect. Also, *in vitro* data regarding C18 FA were analyzed considering the weekly runs as random block and the incubation time as categorical fixed effect. When needed, the group option of the repeated statement was included in the model to accommodate the variance heterogeneity. *In vitro* data regarding the C20 FA were analyzed using linear, quadratic, and cubic polynomial orthogonal contrasts after computing the contrast coefficients with the Proc IML of SAS. Least square means and standard error of the mean are reported and treatment or contrast effects were considered significant at *P* < 0.05.

### Data availability

Most of the data generated or analyzed during this study are included in this published article and its supplementary information files. Any other remaining necessary data is available from the corresponding author upon reasonable request.

## Electronic supplementary material

Supplementary Information
