# Supplementary material for: Nannochloropsis oceanica, a novel natural source of rumen-protected eicosapentaenoic acid (EPA) for ruminants
Source: Sci Rep. 2018 Jul 6;8:10269. doi: 10.1038/s41598-018-28576-7 (PMC6035222; doi:10.1038/s41598-018-28576-7)
Supplement: Supplementary file 1 — Supplementary Information [file 41598_2018_28576_MOESM1_ESM.pdf]

Nannochloropsis oceanica, a novel natural source of rumen-protected eicosapentaenoic acid (EPA) for ruminants

Susana P. Alves[1], Sofia H. Mendonça[2], Joana L. Silva[2] & Rui J.B. Bessa[1]

[1]CIISA - Centro de Investigação Interdisciplinar em Sanidade Animal, Faculdade de Medicina Veterinária, Universidade de Lisboa, Av. da Universidade Técnica, 1300-477 Lisboa, Portugal

[2]ALLMICROALGAE, Av. Eng. Duarte Pacheco 19, 9º piso, 1070-100 Lisboa, Portugal

Supplementary material is presented below

Table S1: Effect of EPA-sources on fatty acids (< 18 carbon-atoms) and dimethyl acetals (DMA) concentration (µg/tube) at 24 hours of in vitro batch incubation with strained rumen fluid.

Item	Control	EPA sources[2]				*P* value
		EPA	*N. oceanica* SD	*N. oceanica* FD	*P. tricornutum* FD	
Fatty acids						
10:0	3.2^{c} ± 0.80	5.3^{bc} ± 0.72	7.0^{ab} ± 0.72	8.3^{a} ± 0.72	7.5^{ab} ± 0.72	<0.001
12:0	65.7 ± 11.99	68.5 ± 11.49	77.5 ± 11.49	78.8 ± 11.49	92.0 ± 11.49	0.044
i-13:0	4.1 ± 1.19	3.8 ± 1.01	6.3 ± 1.01	7.3±1.01	4.5 ± 1.01	0.112
13:0	4.0 ± 0.73	3.8 ± 0.62	2.8 ± 0.62	3.3 ± 0.62	3.5 ± 0.62	0.692
14:0	79.0^{d} ± 2.00	83.8^{d} ± 3.17	107^{c} ± 2.0	136^{b} ± 2.9	171^{a} ± 2.3	<0.001
i-15:0	16.2 ± 1.41	16.3 ± 1.24	19.5 ± 1.24	18.0 ± 1.24	19.8 ± 1.24	0.090
a-15:0	35.2 ± 1.12	35.8 ± 1.00	37.8 ± 1.00	36.3 ± 1.00	37.0 ± 1.00	0.239
15:0	27.3^{c} ± 1.29	30.8^{bc} ± 1.18	33.0^{b} ± 1.18	33.5^{b} ± 1.18	37.5^{a} ± 1.18	<0.001
i-16:0	14.4 ± 1.31	14.0 ± 1.28	15.5 ± 1.28	14.8 ± 1.28	15.0 ± 1.28	0.260
16:0	864^{c} ± 63.6	868^{c} ± 63.0	1111^{b} ± 63.0	1261^{a} ± 63.0	1295^{a} ± 63.0	<0.001
16:1n-7	4.0^{c} ± 1.16	5.5^{c} ± 0.87	56.3^{b} ± 4.27	239^{a} ± 5.09	47.5^{b} ± 4.03	<0.001
16:2n-4	n.d.	n.d.	n.d.	n.d.	9.1 ± 2.42	-
16:3n-4	n.d.	n.d.	n.d.	n.d.	23.7 ± 3.30	-
16:4n-1	n.d.	n.d.	n.d.	n.d.	6.0 ± 2.01	-
i-17:0	9.0 ± 1.24	10.5 ± 1.36	12.0 ± 1.27	9.8 ± 1.20	12.8 ± 1.95	0.338
a-17:0	19.2 ± 1.36	19.8 ± 1.29	18.5 ± 1.29	18.0 ± 1.29	18.8 ± 1.29	0.487
17:0	18.7 ± 3.31	20.8 ± 2.97	23.0 ± 2.97	23.3 ± 2.97	25.8 ± 2.97	0.347
Total BCFA	97.8 ± 4.34	100 ± 4.0	109 ± 4.0	104 ± 4.0	108 ± 4.0	0.517
Dimethyl acetals (DMA)						
12:0	4.7 ± 0.96	4.0 ± 0.85	5.3 ± 0.85	4.3 ± 0.85	2.3 ± 0.85	0.091
i-14:0	5.3 ± 0.56	6.0 ± 0.49	6.0 ± 0.49	6.8 ± 0.49	5.0 ± 0.49	0.114
14:0	12.7 ± 1.53	10.8 ± 1.50	12.0 ± 1.50	12.5 ± 1.50	11.5 ± 1.50	0.071
i-15:0	6.3 ± 1.25	4.0 ± 1.05	5.7 ± 1.05	6.3 ± 1.05	3.3 ± 1.05	0.124
a-15:0[1]	28.1 ± 17.76	23.7 ± 18.36	28.5 ± 21.38	51.1 ± 20.87	38.1 ± 20.87	0.697
15:0	4.7 ± 0.43	4.0 ± 0.36	4.5 ± 0.36	4.5 ± 0.36	3.3 ± 0.36	0.107
16:0	32.6 ± 2.68	30.5 ± 2.52	31.0 ± 2.52	31.8 ± 2.52	29.0 ± 2.52	0.534
18:0	1.4 ± 0.63	2.0 ± 0.60	2.5 ± 0.60	2.5 ± 0.60	2.0 ± 0.60	0.250
*c*9-18:1	5.1^{ab} ± 1.00	4.5^{ab} ± 0.86	5.3^{ab} ± 0.86	6.0^{a} ± 0.86	2.0^{b} ± 0.86	0.032
*c*11-18:1	4.3 ± 0.33	3.8 ± 0.25	3.8 ± 0.63	3.8 ± 0.25	2.8 ± 0.63	0.504
Total DMA	106 ± 15.5	78.8 ± 13.43	83.0 ± 15.51	98.0 ± 13.43	74.3 ± 13.43	0.510

[1] Co-elutes with a non-fatty acid compound.

[2] EPA sources: EPA (free FA), *Nannochloropsis oceanica* dehydrated using spray-dried (SD) or freeze-dried (FD), and *Phaeodactylum tricornutum* dehydrated using freeze-dried (FD).

n.d. not detected

Different letter superscripts (a, b, c) within rows indicate statistically significant ($P < 0.05$) differences between means.

Table S2: Effect of incubation time on C20 fatty acids (µg/tube) during 0 to 24 hours of in vitro batch incubation with strained rumen fluid and a total mixed ration supplemented with EPA, *Nannochloropsis oceanica* dehydrated using spray-dried (SD) or freeze-dried (FD), and *Phaeodactylum tricornutum*.

Item	Incubation time (hours)					SEM	Contrasts[2]		
	0	2	4	10	24		L	Q	C
**EPA (as free FA)**									
20:0	18.8	18.8	21.7	35.0	68.8	4.22	<0.001	0.227	0.467
Total 20:1	1.5	2.0	7.2	36.3	55.5	5.93	<0.001	0.128	0.156
Total 20:2	n.d.	n.d.	3.3	15.0	9.3	1.57	<0.001	<0.001	0.026
Total 20:3	n.d.	n.d.	15.6	16.8	3.5	3.64	0.690	0.001	0.528
Total 20:4	n.d.	36.3	37.8	13.0	5.0	3.76	0.003	0.007	<0.001
Total 20:5[1]	n.d.	7.75	1.67	n.d.	n.d.	0.94	0.015	0.565	0.005
EPA	181	87.3	65.5	28.5	22.3	10.23	<0.001	<0.001	<0.001
***Nannochloropsis oceanica* (SD)**									
20:0	18.0	18.8	22.0	43.3	87.5	4.70	<0.001	0.343	0.247
Total 20:1	1.5	4.3	8.3	33.8	43.3	3.35	<0.001	0.001	0.016
Total 20:2	n.d.	n.d.	4.0	4.3	4.3	0.98	-	-	-
Total 20:3	6.8	10.8	10.8	8.0	4.0	1.16	0.001	0.048	0.011
Total 20:4	37.0	36.3	35.3	26.5	17.3	1.50	<0.001	0.237	0.084
EPA	183	150	138	92.3	56.8	6.81	<0.001	<0.001	0.374
***Nannochloropsis oceanic* (FD)**									
20:0	18.5	18.5	21.0	43.8	83.5	4.82	<0.001	0.505	0.114
Total 20:1	3.7	2.2	10.3	36.0	43.8		<0.001	0.020	0.079
Total 20:2	n.d.	n.d.	5.5	6.5	3.3	0.64	-	-	-
Total 20:3	6.8	15.0	18.3	10.5	5.8	1.63	0.002	0.006	<.0001
Total 20:4	88.8	81.3	70.5	53.8	48.8	2.63	<.0001	<.0001	0.939
EPA	226	170	147	134	127	3.9	<0.001	<0.001	<0.001
Phaeodactylum tricornutum									
20:0	19.5	19.5	20.3	25.3	56.5	3.74	<0.001	0.005	0.983
Total 20:1	3.8	4.5	5.8	21.5	49.0	5.95	<0.001	0.654	0.333
Total 20:2	n.d.	n.d.	6.7	11.8	14.5	2.67	-	-	-
Total 20:3	3.0	4.0	8.0	20.3	7.5	4.92	0.211	0.005	0.256
Total 20:4	10.3	26.0	30.3	20.0	10.3	1.70	<0.001	<0.001	<0.001
Total 20:5[1]	n.d.	4.3	3.5	1.0	n.d.	0.32	-	-	-
EPA	201	143	117	76.3	34.5	5.12	<0.001	<0.001	<0.001

[1] without EPA

[2] Contrasts: linear (L), quadratic (Q), or cubic (C) components of the response to incubation time.

n.d. not detected

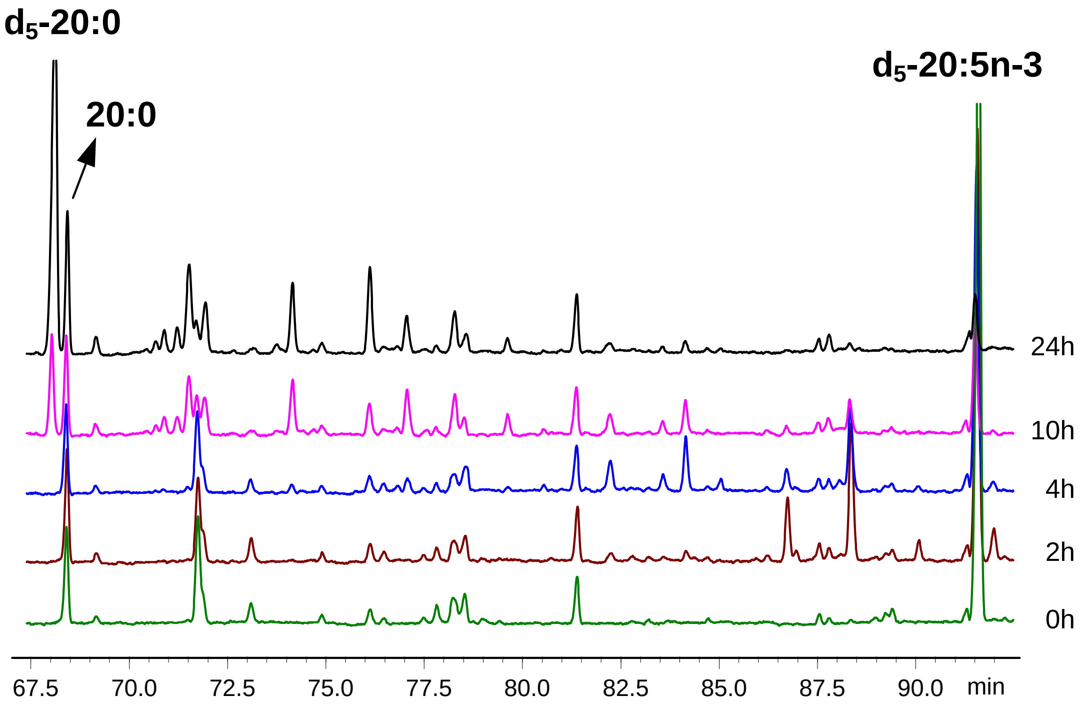

Figure S1. Partial GC-MS chromatograms from the region between 68 and 93 min of in vitro batch incubations with strained rumen fluid and a total mixed ration supplemented with deuterated EPA (d_5-20:5n-3) at 0, 2, 4, 10 and 24 hours (from bottom to the top).
